# Linking brain structure and activation in anterior insula cortex to explain the trait empathy for pain

**DOI:** 10.1002/hbm.24858

**Published:** 2019-11-05

**Authors:** Yun Li, Tingting Zhang, Wenjuan Li, Junjun Zhang, Zhenlan Jin, Ling Li

**Affiliations:** ^1^ Key Laboratory for NeuroInformation of Ministry of Education, High‐Field Magnetic Resonance Brain Imaging Key Laboratory of Sichuan Province, Center for Psychiatry and Psychology, School of Life Science and Technology University of Electronic Science and Technology of China Chengdu China 610054; ^2^ School of Management Chengdu University of Traditional Chinese Medicine Chengdu China 611137

**Keywords:** anterior insula cortex, empathy for pain, gray matter volume, trait empathy

## Abstract

The ability to perceive, understand, and react to the feelings of others' pain is referred to as empathy for pain which is composed of two components, affective‐perceptual empathy and cognitive‐evaluative empathy. Recent reviews on the neural mechanisms of empathetic pain showed the anterior insula (AI) cortex as a core circuit for empathy. However, little is known about the modulation of brain anatomy and empathic responses by trait measures of empathy (trait empathy). Thus, we investigated whether individual variation in the personality trait of empathy is associated with individual variation in the structure of specific brain regions using voxel‐based morphometry (VBM). We further investigated the relationship between the trait empathy and the activity of the same regions using state measures of empathy for pain in a trial‐by‐trial fashion in the given situation. VBM analysis indicated a small but significant negative relationship between trait empathy and gray matter volume in the bilateral AI. Functional MRI study further demonstrated that experimentally induced activity of the bilateral AI during state empathy for pain was also correlated with trait empathy. An asymmetry exists between the right and left AI between the affective and cognitive empathy. The right AI was found to be involved in the affective‐perceptual form of empathy and the left AI was active in cognitive‐evaluative forms of empathy. The interindividual differences in trait empathy may be reflected both in the state empathy and more stable brain structure difference.

## 1 INTRODUCTION

Pain can be experienced by self or perceived in others, which is a special psychological state with great evolutionary significance (Jackson, Meltzoff, & Decety, 2005). The ability to identify and share others' feelings and experiences of pain is known as empathy for pain in some literature (Coll et al., 2017; Decety, Jackson, & Brunet, 2007; Hillis, 2014). Although the definition of empathy for pain differs from study to study, it can be broadly defined as experiencing a painfully affective or sensory state by a perceived individual, which consists of two forms: the “affective‐perceptual” empathy and the “cognitive‐evaluative” empathy. The current empathy for pain studies postulated that empathy can be automatically induced in participants by observation without knowing the purpose of the experiment, which tests the “affective‐perceptual” empathy (Fan, Duncan, de Greck, & Northoff, 2011). In contrast, other studies have suggested that “cognitive‐evaluative” empathy are substantially influenced by whether or not one attends to watch, hear or imagine another person's state of actual pain or a potentially threatening state of tissue injury through the explicit imagination or evaluation of feelings (Canizales, Voisin, Michon, Roy, & Jackson, 2013; Vachon‐Presseau et al., 2012; Vistoli, Achim, Lavoie, & Jackson, 2016). As a complex social psychological phenomenon, empathy for pain can help people avoid risks and danger, establish good interpersonal relationships, and promote prosocial behaviors (Danziger, Faillenot, & Peyron, 2009; Enzi, Amirie, & Brüne, 2016; Masten, Morelli, & Eisenberger, 2011; Wu et al., 2017).

Because there is no appropriate device that can objectively measure empathy, some researchers have relied on self‐reported psychometric scales of empathy (e.g., Interpersonal Reactivity Index [IRI]; Davis, 1983), that refer to the situation‐independent affective‐perceptual and cognitive‐evaluative processes contributing to empathy experience (Avenanti, Minio‐Paluello, Bufalari, & Aglioti, 2009). Following growing interest from social neuroscientists, the neural underpinnings of brain structure and activation to explain trait empathy for pain has become a topic of intensive research.

Previous image‐based and coordinate‐based meta‐analysis indicated that a core network consisting of bilateral anterior insular cortex (AI) was associated with empathy for pain (Keysers & Gazzola, 2014; Lamm, Decety, & Singer, 2011; Zaki & Ochsner, 2012). Results from case and group studies found that the volumes of the AI injury were significantly associated with trait empathy. A lesion‐symptom mapping study of veterans who had brain injuries found that damage in insula was associated with reductions on affective trait empathy (Driscoll, Dal Monte, Solomon, Krueger, & Grafman, 2012). Another study tested 27 patients with acute infarction also found that impairment of affective trait empathy was associated with infarcts in the AI (Leigh et al., 2013). On the contrary, recent studies have yielded different results. A case report found that a patient with left insula damage showed preserved ability to recognize facial emotional expressions but experienced difficulties on executive functions (Švegar, Antulov, Tkalčić, & Antončić, 2016). Actually, the findings of lesion studies are mixed, and these observations do not provide causal evidence for the respective roles of anterior insular in trait empathy.

The stable personal characteristics (trait empathy) may also reflect the on‐line effect of the observed painful stimuli (state empathy). The current paradigms of neuroscience (implicit/explicit empathy for pain paradigms) postulate that watching, hearing or imagining another person's state of actual pain or a potentially threatening state of tissue injury tests the state empathy for pain (Avenanti et al., 2009; Jackson et al., 2005). While trait empathy measures reflect participants' stable dispositions, state empathy measures are more situation‐dependent and directly linked to the observed painful stimuli. The affective‐perceptual component related to pain perception is known to be encoded by the AI, which is involved in representing and integrating internal and emotional feeling states (Craig, 2002b). An empathy training study found that receiving help elicited a classical learning signal in the AI, and this signal in turn predicted a subsequent increase in state empathy (Hein, Engelmann, Vollberg, & Tobler, 2016). Previous functional MRI (fMRI) studies also correlated the trait empathy with brain activities to empathy for pain. For example, Singer et al. found that affective empathy scores of healthy adults (captured by a subscale of the IRI) were positively correlated with the percentage signal change responses to empathy‐for‐pain in the left insula (Singer et al., 2004). However, while changed in visual perspective of empathy‐for‐pain, only the right AI were positively correlated to affective‐perceptual component of empathy (captured by a subscale of the IRI) (Vistoli et al., 2016). Another study found that the perception and assessment of others' pain was associated with significant bilateral activities in the anterior insula, but no activity was correlated with the IRI scores (Jackson et al., 2005).

Although, there is ample evidence relating individual differences in self‐reported trait empathy to local cortical gray matter volume (GMV) and implicit neural activity. Structural correlations of certain cognitive functions may vary between patients and healthy people, and fMRI does not allow for causal inferences, only correlations. Thus, observing structural and functional associations of the AI with trait empathy for pain in healthy people may provide useful information.

In the present study, we conducted both a voxel‐based morphometry (VBM) and a functional imaging study. We used the revised Chinese version of the IRI to measure trait empathy. In addition, a well‐known physical pain observation task was used to induce state empathy for pain. Finally, we combined the above structural and functional brain imaging results to reveal the biological mechanisms underlying the structure and function of the AI and the individual differences of trait empathy. We assumed that the GMV of the left and right AI was related to the trait empathy. Additionally, individual trait empathy was associated with the activity in the left and right AI.

## 2 MATERIALS AND METHODS

### 2.1 Participants

We recruited 26 right‐handed volunteers (12 females; mean age 22.8 ± 2.1 years) from the University of Electronic Science and Technology of China to participate in this study. Owing to technical problems (task fMRI scanning failure), the structural MRI data from 26 participants were included in the VBM analyses and the functional MRI and behavioral data from 22 participants were included in the subsequent fMRI analyses and behavioral analyses. All the participants had no history of head injury, neurological problems, prolonged pain, diagnosed psychiatric disorder, regular medication of any kind and magnetic object in the body, and had normal or corrected‐to‐normal color vision. Each participant signed an informed consent form before the experiment. The local committee for the Protection of Human Subjects for the University of Electronic Science and Technology of China approved this study. The methods were carried out in accordance with the approved guidelines and all experiments conformed to the Declaration of Helsinki (World Medical Association, 2013). After the experiment, all participants received monetary compensation for their time and effort.

### 2.2 Assessment of trait empathy

The measurements of empathic traits reflect stable personal characteristics that individuals may have for different types of situations. Before scanning, participants completed the Chinese version of Davis' Interpersonal Reactivity Index IRI (IRI‐C) (Zhang, Dong, Wang, Zhan, & Xie, 2010) in a quiet testing room using the Wenjuanxing (https://www.wjx.cn/) website. The IRI‐C consists items which can be answered on a five‐point Likert scale ranging from 1 (“Does not describe me well”) to 5 (“Describes me very well”). The items provide individual scores on four subscales (PT: Perspective Taking Scale; FS: Fantasy Scale; EC: Empathy Concern Scale; PD: Personal Distress) to assess personal traits associated with the cognitive and affective processes contributing to empathy experience. PT and FS assess cognitive components of trait empathy, while EC and PD assess affective trait empathy, respectively (Chiang, Hua, Tam, Chao, & Shiah, 2014; Davis, 1983). Higher scores on the EC, PT, and FS scales are associated with higher capacity for empathy. In line with previous studies (Melchers, Montag, Reuter, Spinath, & Hahn, 2016; Rankin et al., 2006), we decided to focus our analysis on IRI‐C subscales scores rather than on total scores. Cronbach's alpha for IRI‐C was 0.873.

### 2.3 Experimental stimuli

**Figure 1:**
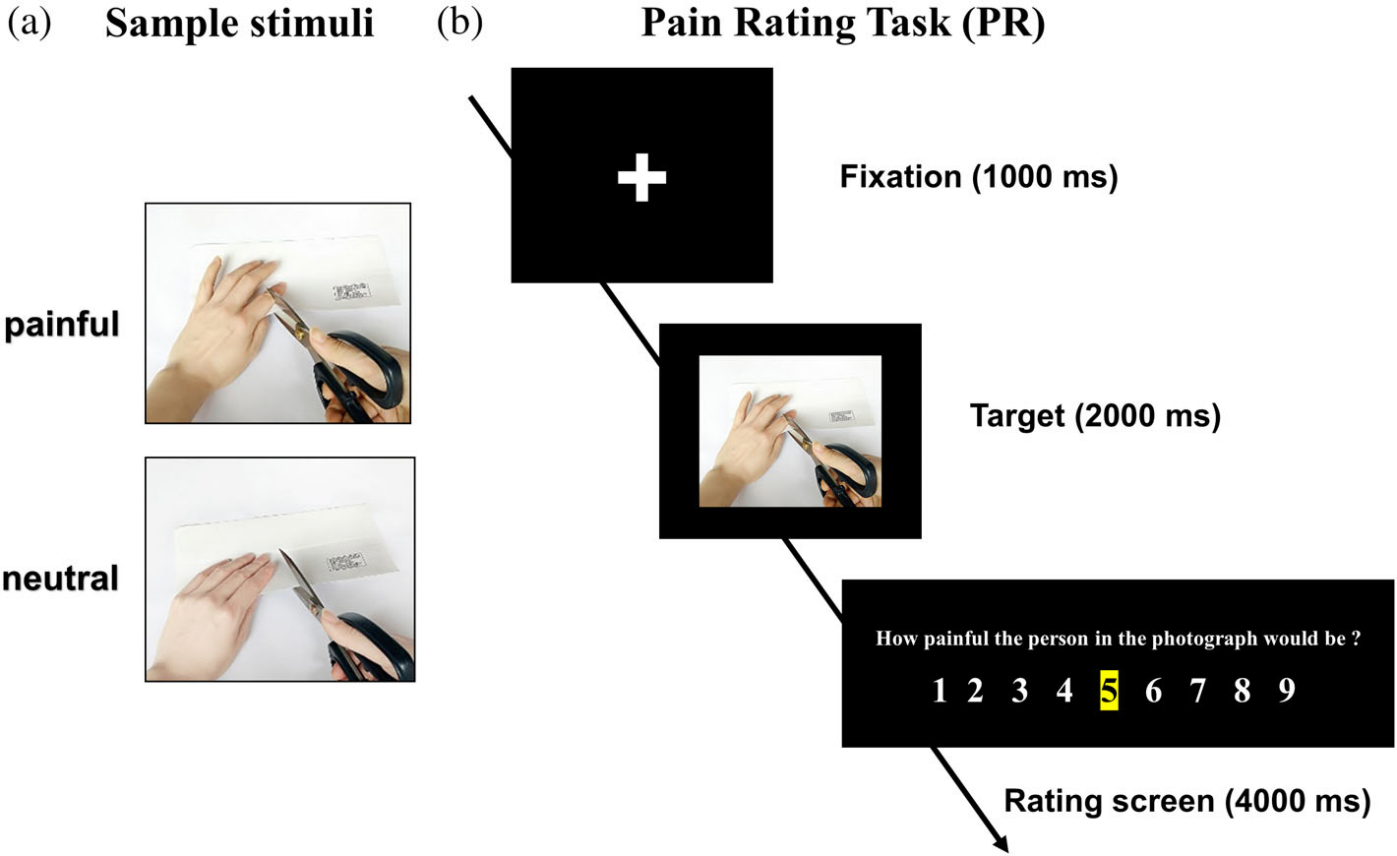
(a) Sample stimuli. (b) The Pain Rating Task in experiment. Participants viewed images of others in painful and neutral situations and indicated how painful the person in the image was suffering from pain

A set of 108 still digital color photographs showing another person's hand or foot in painful or neutral situations were shot from angles promoting first‐person perspective (less mental rotation of the limb required for the participants), which are similar to the stimuli used in previous studies (Gu et al., 2010). After the initial screening, the degree of pain expressed in the images was estimated by an independent group of 67 college students based on a 9‐point scale from 1 (not painful at all) to 9 (extremely painful). The pain ratings of painful and neutral photographs (mean values ± *SD*: 6.85 ± 1.35 and 1.57 ± 0.31, respectively) were significantly different (*t*_(66)_ = 24.85, *p* < .000). Photographs with estimated pain scores higher than 6 points and lower than 2 points eventually were selected as the experimental materials. Finally, a total of 64 photographs were included in this study. Half of the photographs showed painful events and the other half showed neutral events that were identical in physical properties (i.e., context, size, background, brightness, contrast). All photographs (640 × 480 pixels) had a good degree of differentiation and can be used in formal experiments (see Figure 1a).

### 2.4 Scanning method and procedure

Sixty‐four stimuli were presented in a blocked fMRI design following Kao's instructions (Kao, 2014). Participants took part in a one sequential fMRI session consisted of 16 blocks and 2 conditions (painful/neutral). Each block consisted of four 7‐s trials of the same condition (fixation screen = 1.0 s, target screen = 2.0 s, rating screen = 4.0 s), and each stimuli was followed by a visual analogue rating scale ranging from 1 (“not painful at all”) to 9 (“extremely painful”). The order of conditions was pseudorandomized according to the evoked pain (painful or neutral). No photograph was presented more than once throughout the whole experiment. A blank screen of 7 s was inserted between each block of trials to allow skin conductance and hemodynamic responses to return to baseline. Participants were instructed to rate the intensity of pain they thought the person would feel in each situation (Pain Rating Task [PR]) (see Figure 1b). For each trial, the initial cursor was at “5” so that every trial in every condition required moving the cursor along the rating scale by pressing and holding down either of two keys, thereby controlling for the motor output involved in the rating process across all conditions. Participants were provided with several training trials prior to the scanning sessions in order to learn to use the rating scale and perform the task accurately within the allotted time. The completely scanning process took a total of 10 min. After scanning, participants debriefed about how they felt during the experiment, and some questions concerning what strategy they used during the task.

### 2.5 MRI data acquisition

All participants underwent fMRI in a 3.0T GE DISCOVERY MR750 scanner (General Electric Medical System, Milwaukee, WI) using an 8‐channel phased array head coil. Functional images were collected by a single shot, gradient recalled echo‐planar imaging (EPI) sequences (TR = 2000 ms, TE = 30 ms, flip angle = 90°, matrix size = 64 × 64, voxel size = 3.75 × 3.75 × 3 mm^3^; 43 slices oriented in an AC‐PC line). During the 6.5 min resting state fMRI scan, participants were instructed to hold still, close their eyes, and relax their minds. High‐resolution T1‐weighted images were acquired by using a dimensional fast spoiled gradient echo (T1‐3D FSPGR) sequence (TR = 5.932 ms, TE = 1.956 ms, flip angle = 9°, matrix size = 256 × 256, FOV = 25.6 × 25.6 cm^2^, and slice thickness = 1 mm) to control for any anatomic abnormalities and increase normalization accuracy during preprocessing.

### 2.6 Voxel‐based morphometry

Anatomical brain images were analyzed with CAT12 toolbox (http://dbm.neuro.uni-jena.de/cat/) which was incorporated into SPM12 (Statistical Parametric Mapping; Wellcome Trust Centre for Neuroimaging, London, UK, http://www.fil.ion.ucl.ac.uk/spm) running under MATLAB R2013a (MathWorks, Sherborn, MA). Structural MRI images were tissue classified, and gray matter, white matter and cerebrospinal fluid segments were saved. A study‐specific template was generated using the inbuilt DARTEL algorithm, and the warping functions generated by DARTEL were used to spatially normalize the gray matter segments and modulate them by the Jacobian determinant. Finally, the normalized and modulated gray matter segments were smoothed using an 8 mm full width at half‐maximum (FWHM) Gaussian kernel. We used the default parameters of CAT12. The preprocessed images were entered into a multiple regression model with all subscales included in the same design matrix to identify cortical regions that showed a correlation with the subscales of the IRI‐C. We included age and total gray matter as covariates of no interest in the design matrix to regress out any effects attributable to them. We performed whole‐brain analyses and investigated both positive and negative correlation between scale value and GMV. Significant GMV associated with the trait empathy scores were identified using a threshold of *p* < .05 (uncorrected for multiple comparisons).

### 2.7 MRI data analysis

MRI data preprocessing was performed using SPM12 (Statistical Parametric Mapping; Wellcome Trust Centre for Neuroimaging, London, UK, http://www.fil.ion.ucl.ac.uk/spm), implemented in MATLAB R2013a (MathWorks, Sherborn, MA). The first five EPI volumes of the fMRI images were discarded for signal stabilization. fMRI data preprocessing included slice timing correction, three‐dimensional motion correction, coregistration to individual anatomical images, normalization to the Montreal Neurological Institute (MNI) reference space (3 × 3 × 3 mm^3^), and spatial smoothing with an 8 mm Gaussian kernel (full width at half‐maximum). One session from one subject with a total vector motion >1.5 mm or rotation >1.5° was excluded from further analysis. The fixation and rating phases were considered covariates of no interest to partial out their contribution to brain activation in the single‐subject analyses. In addition, the six motion parameters were also modeled as effects of no interest. A first level of analysis was computed subject‐wise using the general linear model with hemodynamic response function modeled as a boxcar function whose length covered the four successive pictures of the same condition (painful or neutral). First‐level contrasts were introduced in second‐level random‐effect analysis to allow for population inferences. At the group level, we first carried out paired *t* test to evaluate different activations between the painful condition and the neutral condition (painful‐neutral) and vice versa (neutral‐painful). Then, the following whole‐brain multisubject analysis was conducted by using a random effects model with a one‐sample *t* test on the summary statistic. The statistical contrast maps were thresholded at *p* < .05 (corrected for false discovery rate [FDR]) to control for multiple comparison. For the pain‐related EPIs, contrasts were made between the pain conditions taken together and the neutral conditions (Forman et al., 1995).

Regions of interest (ROIs) of the bilateral AI were defined based on the multisubject statistical maps. A 9‐mm radius sphere (centered on the peak activation of each cluster) was drawn as a ROI using the MarsBaR toolbox (http://marsbar.sourceforge.net). The blood oxygenation level‐dependent signal change (BOLD) of each ROI was then calculated for each condition (painful/neutral), using the mean signal intensity of each ROI.

### 2.8 Data analysis

Rating scores and reaction times (RTs) were measured. Furthermore, the mean rating scores and mean RTs were evaluated. Mean values ± *SD* was reported for the behavioral results. The relationships between trait empathy scores and the blood oxygenation level‐dependent signal change (BOLD) and GMV were analyzed with Pearson's correlation coefficients. All significance tests were two‐tailed, and *p*‐values were set at .05. All the statistical analysis was performed using IBM SPSS Statistics 21 (IBM, New York).

## 3 RESULTS

### 3.1 Trait empathy (IRI‐C) results

In order to compare the results of our study with published norms, we first calculate descriptive statistics and normality tests (see Table 1). Skewness and kurtosis for the IRI‐C subscales were almost close to 0 and the Kolmogorov–Smirnov test showed that the scores were normally distributed. We were unable to replicate differences between male and female scores on all four subscales of the IRI‐C. However, a marginally significant gender differences between male and female were found on the EC (*t*_(24)_ = −1.894, *p* = .070) and the FS (*t*_(24)_ = −1.979, *p* = .059) subscales.

**Table 1:** IRI‐C descriptive statistics

	PT	EC	FS	PD
*N*	26	26	26	26
Mean	3.599	3.780	3.417	3.200
*SD*	0.416	0.482	0.560	0.635
Skewness	0.257	0.284	0.287	0.349
*SE* of skewness	0.456	0.456	0.456	0.456
Kurtosis	−0.559	−0.280	0.751	−1.354
*SE* of kurtosis	0.887	0.887	0.887	0.887
Kolmogorov–Smirnov *Z*	0.723	0.657	0.498	1.005
*p* value (two‐tailed)	0.673	0.781	0.965	0.264

Abbreviations: EC, Empathy Concern Scale; FS, Fantasy Scale; IRI‐C, the Chinese version of the Interpersonal Reactivity Index; PD, Personal Distress Scale; PT, Perspective Taking Scale.

### 3.2 Behavioral results

**Figure 2:**
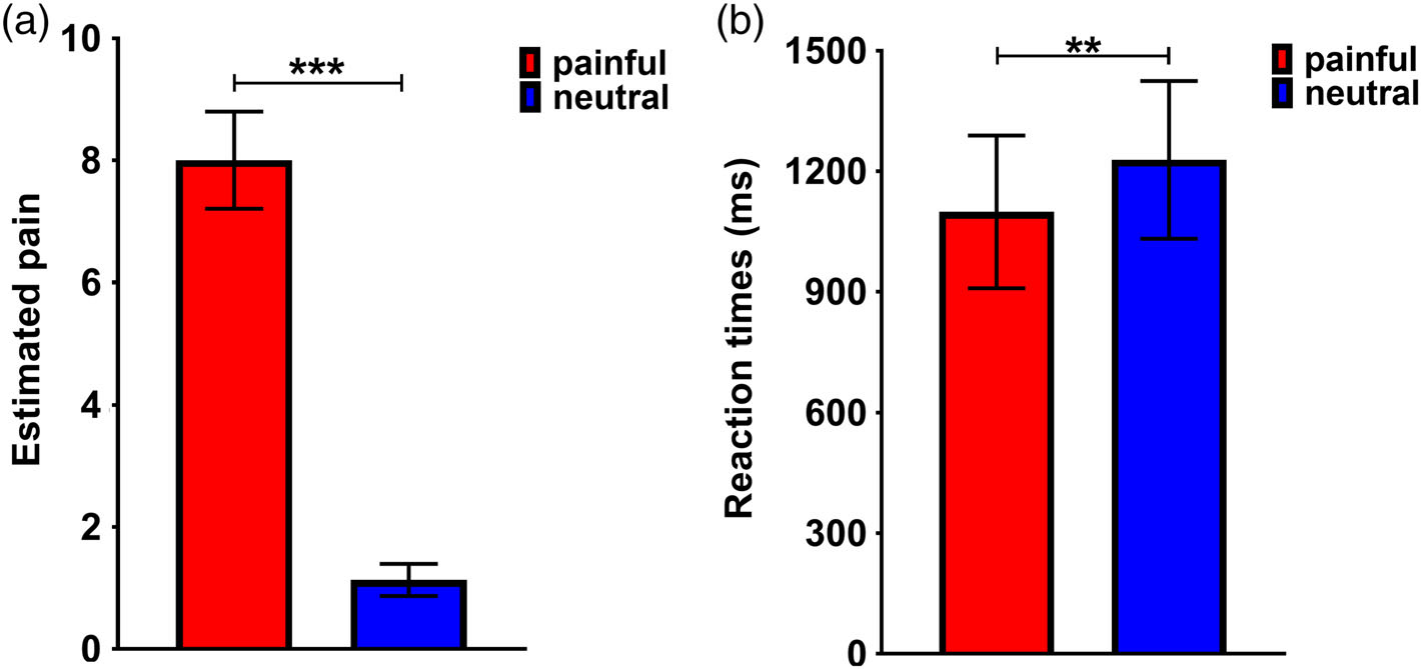
The behavioral data in the fMRI experiment. (a) Bar charts depicting mean estimated pain rating difference scores per condition for painful and neutral photographs. (b) Bar charts depicting mean reaction times per condition for participants rating painful and neutral photographs; Error bars depict *SD* (***p < .001, **p < .01, *p < .05)

Mean rating scores of the two experimental conditions (painful and neutral) were 8.003 ± 0.795 and 1.133 ± 0.263 (mean ± *SD*), respectively (see Figure 2a). A paired *t* test on the mean rating scores revealed significant differences (*t*_(21)_ = 35.531, *p* < .000). Although the participants were not asked to react quickly, we recorded the reaction time when they scored. Mean RTs for painful and neutral conditions was 1,098.887 ± 190.212 and 1,228.568 ± 196.339 (mean ± *SD*) respectively (see Figure 2b), and a paired *t* test on the mean RTs revealed significant differences (*t*_(21)_ = −2.891, *p* = .009). Postscan interviews confirmed that most participants reported imagining the painful situations occurring to other people. Note that the mean rating scores for painful condition was correlated with mean RTs for the pain scenarios (*r*_(22)_ = 0.508, *p* = .016).

### 3.3 GMV of the AI correlated with trait empathy

**Figure 3:**
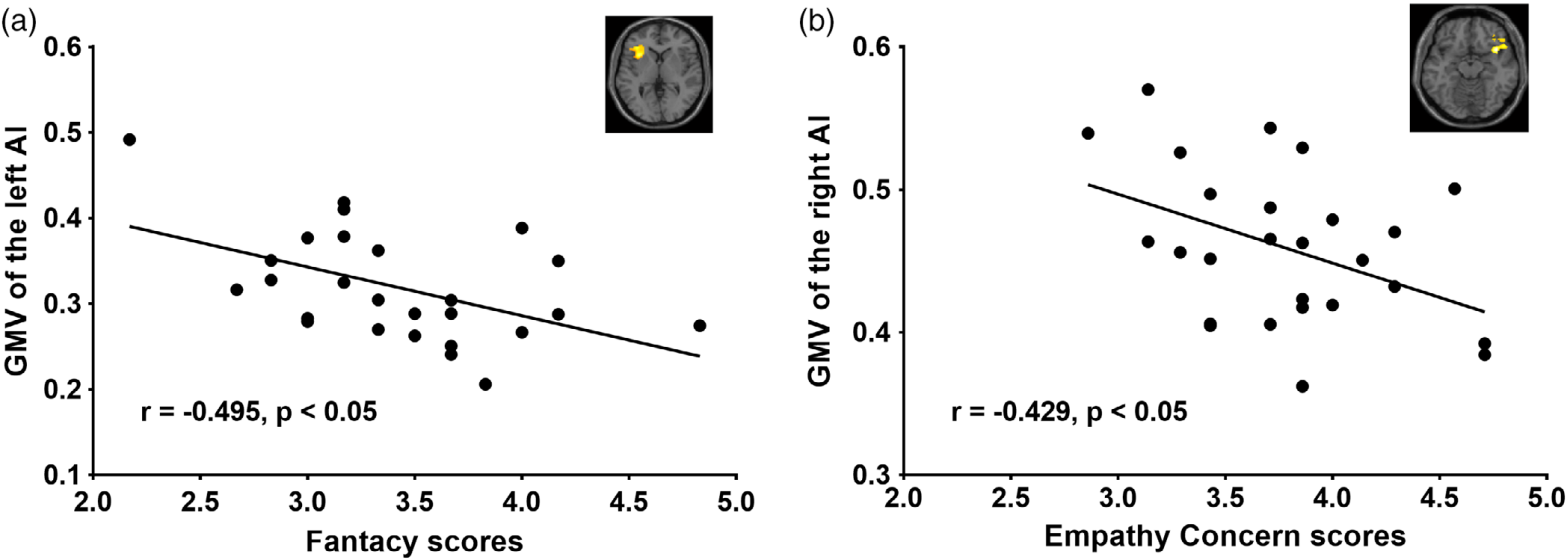
Association between GMV of the AI and trait empathy. Scatter plot demonstrating the association between GMV of the left and right AI and trait empathy (at a threshold of *p* < .05 uncorrected for multiple comparisons) for the whole‐brain volume analysis. AI, anterior insula cortex; GMV, gray matter volume

In this study, we first investigated the association between GMV of the AI and trait empathy scores. As depicted in Figure 3 and Table 2, there was a negative correlation between trait empathy scores and the GMV of the AI. In a whole‐brain analysis of anatomical brain images, we found a significant negative relationship between scores on the FS subscale and the GMV of the left AI (*r* = −0.495, *p* = .010; peak coordinates: *x* = −33, *y* = 17, *z* = 0; *t* = 3.34, *p* = .001). Additionally, we found a significant negative relationship between scores on the EC subscale and the GMV of the right AI (*r* = −0.429, *p* = .029; peak coordinates: *x* = 41, *y* = 15, *z* = −14; *t* = 3.16, *p* = .003). We did not observe any significant positive relationship association between GMV of the insula cortex and trait empathy scores.

**Table 2:** Whole‐brain structural analysis examining cortical regions related to scores on each IRI‐C subscale

IRI‐C	Anatomical location	*R* value	*p* value	MNI coordinates			*T* value	*p* value
				*x*	*y*	*z*		
EC	Right insula	**−0.429**	**.029**	**41**	**15**	**−14**	**3.16**	**.003**
	Right IFG	−0.484	.012	44	15	24	2.57	.009
	Right precuneus	−0.676	.000	14	−63	45	3.56	.001
	Right MFG	−0.473	.015	33	20	23	2.45	.011
	Right MCC	−0.445	.023	5	−8	32	2.02	.027
	Right amygdala	−0.415	.035	21	2	−18	2.15	.022
FS	Left insula	**−0.495**	**.010**	**−33**	**17**	**0**	**3.34**	**.001**
	Right IFG	−0.470	.015	48	21	29	3.90	.000
	Right MFG	−0.416	.034	42	50	29	3.48	.001
	Right AG	−0.496	.010	41	−63	54	2.16	.021
	Left SMG	−0.482	.013	−48	−24	24	3.81	.000
	Left IPL	−0.423	.031	−50	−42	38	3.27	.002
	Left IPL	−0.407	.039	−51	−41	38	4.04	.000
	Left MCC	−0.445	.023	−3	−6	33	1.89	.035
PD	Right MFG	−0.388	.050	26	14	50	2.44	.012
	Right AG	−0.400	.043	47	−60	41	2.24	.017
	Right SMA	−0.460	.018	8	−20	71	2.47	.011
	Right precuneus	−0.472	.015	6	−63	72	3.36	.001
	Left SMG	−0.440	.025	−51	−23	23	2.96	.004

*Note*: For each region, we describe: the IRI‐C subscales that correlated with it; the anatomical description of the region; the *R* value and the *p* value; the MNI coordinates of the peak coordinate; the *T* value and the uncorrected *p* value (*p* < .05 uncorrected for multiple comparisons).

Abbreviations: AG, angular gyrus; EC, Empathy Concern Scale; FS, Fantasy Scale; IFG, inferior frontal gyrus; IPL, inferior parietal lobe; IRI‐C, the Chinese version of the Interpersonal Reactivity Index; MCC, mid‐cingulate cortex; MFG, middle frontal gyrus; PD, Personal Distress Scale; SMA, supplementary motor area; SMG, supramarginal gyrus.

The above peculiar inverse relationship not only shows in the insula cortex, but also in other brain regions in whole‐brain analysis (see Table 2). Affective empathic abilities that are oriented toward another person (i.e., EC subscale) were linked with reduced GMV within the right mid‐cingulate cortex (MCC), the right precuneus, the right inferior frontal gyrus (IFG), the right amygdala and the right middle frontal gyrus (MFG); a tendency toward self‐oriented affective empathy (i.e., PD subscale) was linked with reduced GMV in the right middle frontal gyrus (MFG), the right precuneus, the right supplementary motor area (SMA), the right angular gyrus (AG) and the left supramarginal gyrus (SMG), and the ability to empathize with/place oneself into fictional situations (i.e., FS subscale) was associated with decreased GMV in the right middle frontal gyrus (MFG), the right IFG, the right AG, the left MCC, the left SMG and the left inferior parietal lobe (IPL).

### 3.4 Brain activations of the AI correlated with trait empathy

**Figure 4:**
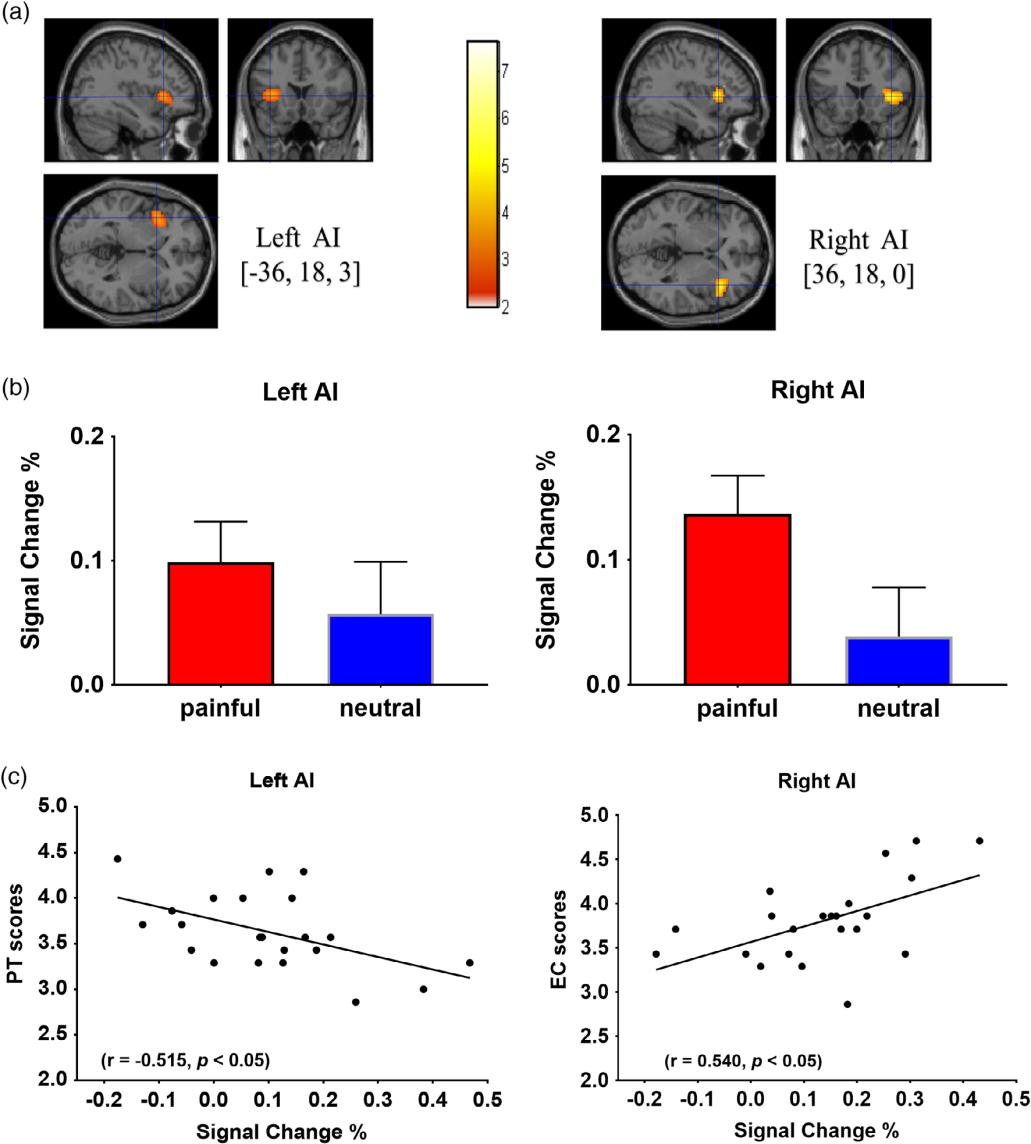
Association between the activity of the AI and trait empathy. (a) The left and right AI clusters significantly activated in the painful condition as compared to the neutral condition. (b) Bar charts depicting the observed event‐related blood oxygenation level‐dependent signal change (%, mean), calculated based on spherical ROIs of 9 mm radius per condition for painful and neutral photographs in the left and right AI. (c) Scatter plot demonstrating association between the trait empathy and the activity of the left and right AI in painful condition. AI, anterior insula cortex; EC scores, score of the Empathic Concern Subscale; PT scores, score of the Perspective Taking Subscale

We performed whole‐brain analysis. Contrasts between painful and neutral conditions (painful > neutral) were first performed to identify regions more activated during perception of others' pain. As expected, increased activation for painful stimuli compared with neutral stimuli was found in the frontal gyrus, occipital visual areas, inferior temporal gyrus, parietal gyrus, cerebellum, and thalamus (see Table 3). There are two brain areas of concern to us. As shown in Figure 4a,b and Table 3, one cluster contained the left AI (peak coordinates: *x* = −36, *y* = 18, *z* = 3; *t* = 3.85, *p* = .009, FDR corrected) and the other cluster consisted of the right AI (peak coordinates: *x* = 36, *y* = 18, *z* = 0; *t* = 4.16, *p* = .006, FDR corrected) were found to be more activated in painful conditions. In an attempt to investigate whether the neural activations of the bilateral AI found in painful condition were correlated with the self‐report trait empathy scores, the plot between the neural activity (the blood oxygenation level‐dependent signals: BOLD) at above coordinates in painful condition and two subscales score showed the significant linear correlation (see Figure 4b,c). The self‐evaluation score of the PT subscale (trait‐cognitive empathy) was negatively correlated with the activity of the left AI (*r* = −0.515, *p* = .014; radius = 9 mm, peak coordinates: *x* = −36, *y* = 18, *z* = 3). The EC scores of trait‐affective empathy was positively correlated with the activity of the right AI (*r* = 0.540, *p* = .010; radius = 9 mm, peak coordinates: *x* = 36, *y* = 18, *z* = 0). No similar association was shown when participants watched the neutral stimuli.

**Table 3:** Whole‐brain analysis: stimulus effects for empathic pain

Region	L/R	MNI coordinates			*V*	*T*
		*x*	*y*	*z*		
*Painful > neutral stimuli*						
Frontal lobe						
Superior frontal gyrus	L	−6	18	42	292	6.29**
Superior frontal gyrus	R	9	24	42	50	5.63**
Inferior frontal gyrus	L	−36	42	6	291	4.14**
Inferior frontal gyrus	R	45	36	24	182	5.90**
Middle frontal gyrus	R	42	48	6	494	4.88**
Precentral gyrus	L	−51	3	24	639	7.62**
Parietal lobe						
Superior parietal gyrus	L	−18	−60	60	438	5.85**
Inferior parietal gyrus	L	−54	−24	48	523	5.37**
Inferior parietal gyrus	R	39	−54	39	235	5.09**
Supramarginal gyrus	L	−54	−24	21	137	4.34**
Supramarginal gyrus	R	57	−30	42	138	3.93**
Postcentral gyrus	L	−36	−27	51	580	6.02**
Postcentral gyrus	R	66	−18	39	70	4.77**
Occipital lobe						
Inferior occipital gyrus	L	−51	−63	−18	223	3.73**
Inferior occipital gyrus	R	42	−69	−12	133	4.34**
Superior occipital gyrus	L	−18	−75	39	120	4.98**
Middle occipital gyrus	L	−30	−87	0	292	6.47**
Middle occipital gyrus	R	33	−78	30	194	5.18**
Temporal lobe						
Inferior temporal gyrus	L	−48	−42	−18	110	3.99**
Inferior temporal gyrus	R	57	−39	−15	174	2.73*
Cerebellum lobe						
Cerebellum	R	18	−54	−24	378	7.07**
Cerebellum	L	−6	−81	−39	48	3.69*
Subcortical						
Insula	L	**−36**	**18**	**3**	**104**	3.85**
Insula	R	**36**	**18**	**0**	**73**	4.16**
Thalamus	L	−15	−24	3	116	4.05**
*Neutral > painful stimuli*						
Frontal lobe						
Middle frontal gyrus	R	27	6	51	104	3.95**
Postcentral gyrus	R	45	−18	51	217	8.89**
Precentral gyrus	R	36	−21	57	290	9.66***
Supplementary motor area	R	9	−6	57	35	5.51**
Parietal lobe						
Angular gyrus	L	−45	−60	24	60	5.32**
Angular gyrus	R	48	−57	39	239	4.43**
Occipital lobe						
Superior occipital gyrus	R	18	−90	18	26	4.82**
Cuneus	L	−9	−90	21	15	4.88**
Cerebellum lobe						
Cerebellum (superior)	L	−15	−54	−21	34	5.93**
Other lobe						
Lingual	L	−15	−81	−9	76	6.54***
Calcarine	R	12	−90	12	31	6.30***

*Note*: Regions included were thresholded by default at *p* < .05, FDR corrected.

Abbreviations: L, the left hemisphere; MNI, the Montreal Neurological Institute coordinates, which reflect the peak of each cluster, not the centroid; *R*, the right hemisphere; *V*, voxel.

*p < .05; **p < .01; ***p < .001.

## 4 DISCUSSION

In the present study, we used VBM analysis combined with a functional MRI study to investigate whether individual differences in self‐reported trait empathy was related to morphological differences and implicit neural responses in human anterior insula cortex. The critical findings were that interindividual differences in trait empathy might be reflected both in the situation‐dependent effect of the state empathy and the situation‐independent brain structure difference. An asymmetry exists between the right and left AI between the affective and cognitive empathy.

As hypothesized, our results showed that individual differences in self‐reported trait empathy were related to morphological differences in the AI. The AI has been noted to be the only region of the brain with consistent associations with all empathy‐related tasks (Mutschler, Reinbold, Wankerl, Seifritz, & Ball, 2013). With its connections to the limbic system and prefrontal cortex, the insula cortex has been implicated as a hub processing and evaluating the emotional elements of pain observed in others (Eres, Decety, Louis, & Molenberghs, 2015; Wylie & Tregellas, 2010). The neuropsychological patient data suggested that lesions to the AI resulted in impairments in trait empathy (Couto et al., 2013; Driscoll et al., 2012; Hillis, 2014; Leigh et al., 2013). Our results further supported the above arguments in terms of the structure of the AI in healthy people and showed consistent findings with previous structural imaging studies that individual differences in brain morphometry in AI were associated with differences in trait empathy (Banissy, Kanai, Walsh, & Rees, 2012; Eres et al., 2015; Mutschler et al., 2013).

Some interesting discrepancies were observed between the present results and our hypothesis. The present investigation revealed an inverse relationship between GMV in the AI and trait empathy; that is, decreased brain volume in the bilateral AI was associated with increased trait empathy scores. Additionally, in a whole‐brain analysis, the peculiar inverse relationship not only show in the AI, but also in other brain regions (e.g., MCC, the precuneus, IFG, the amygdala, MFG, SMA, AG, SMG, and IPL) which is consistent with recent meta‐analyses highlighting these regions as core networks involved in empathy (Fan et al., 2011; Timmers et al., 2018).

Actually, the specific direction of the relationship between trait empathy and the brain morphometry were also found in the mPFC, precuneus, right temporal pole, left IFG, and right superior parietal lobule, which may play a key role in individual differences in other empathy study (Takeuchi et al., 2014). Banissy et al. also found that the GMV of the left AI was negatively correlated with the trait empathy measured by the IRI scale in healthy people (Banissy et al., 2012). VBM studies in other fields have also found a negative correlation between GMV and cognitive performance which may imply that in healthy adults “less is more” (Kanai & Rees, 2011). However, precisely why less GMV in the AI may facilitate empathy is difficult to disentangle. To fully understand this issue, it is necessary to investigate how differences in micro‐structure measurements in insular cortex correlate with individual differences in trait empathy (Banissy et al., 2012).

Except for empathy‐related changes in morphology, the present fMRI data from the same group of participants showed that the activation of the AI was significantly higher when they watched the pain‐related pictures than the activation observed with the neutral pictures. Emma Duerden et al. used quantitative meta‐analytic methods and found that perceiving emotions in others activated bilateral anterior insula (Duerden, Arsalidou, Lee, & Taylor, 2013). Many other studies also highlighted that the AI was a component of the core networks involved in empathy (Allen et al., 2017; Bernhardt & Singer, 2012; Chakrabarti, Bullmore, & Baron‐Cohen, 2006; Jabbi, Swart, & Keysers, 2007; Lamm et al., 2011; Singer et al., 2004). Lesion study provided further evidence that the insula damage may lead to decreased affective response to and decreased withdrawal from painful stimuli. This deficit often means a lack of understanding the meaning of pain (Singh, Giles, & Nasrallah, 2006). This insensitivity to pain is also found in the patient's healthy relatives, which indicates a genetic contribution to the trait (Hooley & Delgado, 2001). These results showed that the activity of the AI is sensitive to pain information (not neutral information) and can affect the state of empathy for pain.

In addition, consistent with previous fMRI studies, inner individual differences in trait empathy were reflected in the activity of the AI. When participants observed the pain‐related images, interindividual differences in trait empathy correlated with the activity in the left and right AI. The PT subscale scores assessing the tendency to spontaneously imagine and assume the cognitive perspective of another person were negatively correlated with the BOLD responses of the left AI. Affective empathetic abilities that are oriented toward another person (the EC subscale scores) were linked with increased activity in the right AI. No similar association was shown when participants watched the neutral stimuli. The AI is traditionally seen as part of a limbic sensory system that encodes and stores representations of the physical state associated with environmental stimuli and represents the emotional self over time (Craig, 2009, 2011; Ernst, Northoff, Böker, Seifritz, & Grimm, 2013; Gu, Hof, Friston, & Fan, 2013; Gu, Liu, Van Dam, Hof, & Fan, 2013; Nieuwenhuys, 2012). The AI, engaged in the conscious representation of emotion in the self and in others through projections to the limbic system, plays a crucial role in emotional and other cognitive functions (Sassa et al., 2012). Individuals with higher trait‐affective empathetic ability have internal sensory systems that are more active. For example, the scores on trait empathy (measured by the IRI scale) could predict the activity of the AI in empathy for pain (Loggia, Mogil, & Catherine Bushnell, 2008). Similar results had been obtained from the study of children. Children were asked to mimic or observe different expressions and the activation of the right AI was correlated with the trait‐affective empathetic ability (Pfeifer, Iacoboni, Mazziotta, & Dapretto, 2008). In addition, self‐reported poor awareness of own and others' feelings was associated with a reduction in the response of insular cortex in autistic and typically developing individuals. This dysfunctional AI connectivity may underlie the emotional and social impairment observed in patients with trait empathy defects (i.e., low in trait empathy) (Caria & de Falco, 2015; Cox et al., 2012; Ebisch et al., 2011). Our findings provide evidence that the AI is sensitive to other's pain information and involved in pain empathy. In other words, activity in the AI affects individuals' judgment of perceived pain information.

However, the AI shows a pronounced asymmetry in its involvement with empathy for pain states. We noticed the correlation between left AI activity and cognitive‐evaluative empathy (measured with the PT subscale of the IRI‐C), the GMV of the left AI and cognitive‐evaluative empathy (measured with the FS subscale of the IRI‐C), and the correlation between affective‐perceptual empathy (from the EC subscale of the IRI‐C) and the activity and GMV in the right AI. Two meta‐analysis (Kober et al., 2008; Wager, Phan, Liberzon, & Taylor, 2003) of insula activation across studies of pain and emotion has found the right AI included in a functional group (lateral paralimbic group, regions such as the thalamus and PAG) which is most closely connected to brainstem and core limbic regions and is functionally related to emotional and affective sensory experience. This functional group has been suggested to be involved in evaluating bottom‐up internal and external affective signals, and then integrating them into motivational states with associated goals (Kober et al., 2008). In particular, previous studies have found that activity in the right AI is involved in the processing signals from the body (Craig, 2002a, 2003, 2009, 2011), which contribute to both broad and specific affective states (Critchley, Wiens, Rotshtein, Öhman, & Dolan, 2004). Therefore, this involvement of the right AI in affective empathy is likely to be related to interaction with subcortical brain areas. On the contrary, cognitive empathy seems to be mediated by left AI. Gu et al. studied the cognition‐emotion functional integration of the AI in empathy for pain. Due to the anatomical and intrinsic functional connectivity with a large scale network of sensorimotor, affective, and cognitive control regions, the left AI serves as a key node in cognition‐emotion information integration process (Gu, Hof, et al., 2013). Particularly, the left AI showed a more robust interaction effect (surviving in both ROI and whole‐brain analyses; Gu, Liu, et al., 2013). The left AI might have advantageous access to cognitive control regions, which makes cognitive empathy seems to be mediated by left AI easier compared with the right AI (Gu et al., 2010, 2015; Gu, Liu, et al., 2013). This asymmetry between the right and left AI might reflect previous findings of functional and anatomical differences in AI (Craig, 2009, 2011).

Furthermore, although consistent with previous findings by Banissy et al., whose studies point to a relationship between the GMV of the left AI and trait empathy values, the study by Banissy and colleagues (Banissy et al., 2012) found a negative correlation between GMV in the left AI and scores in the EC subscale of the IRI. The discrepancy between the study of mine and Banissy et al. may seem not contradictory if it is explained from the asymmetry of the AI. Banissy and colleagues found a negative correlation between local GMV in the left AI and scores in the Empathic Concern Subscale (EC) of the IRI. Furthermore, they found a positive correlation between GMV in the left AI and scores in the Personal Distress Subscale (PD) of the IRI. When these two findings are considered together, the relationship between structural variations in the left AI and specific empathy traits is easy to understand. As a kind of empathic response, PD is defined as a “self‐oriented” feeling of personal unease to another's state, while EC assesses “other‐oriented” tendency to feel sympathy and compassion for others in need (Davis, 1983). In line with this, previous studies found that unlike empathic concerns that promotes a mode of reasoning oriented to improve others' conditions, observing the suffering of another with a prosocial concern and urge to help the suffering person, personal distress fosters a hedonic reasoning which is associated with an aversive, avoidant response that is primarily self‐focused and aimed toward relieving their own distress rather than helping the other person (Carrera et al., 2013; Paciello, Fida, Cerniglia, Tramontano, & Cole, 2013; Thomas, 2013; Zhu et al., 2018). Taking into account this potential characteristic of EC, a negative relationship between scores on the EC subscale of the IRI and brain volume in the left AI and a positive relationship between scores on the PD subscale of the IRI and brain volume in the same region may indicate that more GMV in the left AI is related to higher personal unease to another's state and less tendency to feel sympathy and compassion for others in need. EC and PD both correspond to the notions of other‐oriented and self‐oriented affective trait empathy. That is to say, the left AI might link emotional motivation to behavioral tendencies in empathy. As findings by Gu et al. (Gu, Liu, et al., 2013) suggested that the left AI might be a key node in a neural network that serves as the anatomical basis for cognition–emotion integration. Two meta‐analyses of empathy studies provide indirect supporting evidence that the left AI might be active both in the affective‐perceptual form of empathy and the cognitive‐evaluative form of empathy (Fan et al., 2011; Timmers et al., 2018). The present findings do not confirm that the left AI only plays a role in cognitive empathy. These above arguments remain speculative at present, however.

One possible limitation of the present study might be that we used the first‐person perspective (1PP) and not the third‐person perspective (3PP) to show the experimental materials. Previous findings (Vistoli et al., 2016) showed the activations related to the core network of empathy for pain (i.e., aMCC and insula; Fan et al., 2011) both in the 1PP and 3PP visual perspectives. As previous studies mentioned, to understand another person's visual perspective, one has to transpose the other's spatial image onto the self‐perspective (Harris, 1975; Kessler & Thomson, 2010). Thus, in either 1PP or 3PP visual perspectives, people have to mentally simulate an egocentric visual representation of the context seen. Looking at painful situation from 1PP may be more readily processed than that from 3PP (Basso et al., 2018; Canizales et al., 2013; Vistoli et al., 2016). Some studies also found that painful situations observed in 1PP engage to a greater extent the sensory processes of pain perception comparatively to situations seen from 3PP, which may enhance neurophysiological activity and pain intensity judgments (Canizales et al., 2013; Vistoli et al., 2016). Furthermore, while face‐to‐face interaction with people is common in daily life, some situations may lead to different visual perspective (e.g., the nurse in the hospital). The nurse may need higher empathy (experiencing events through their own eyes or transposition of others' spatial environment onto the self‐perspective, as actors) to better serve their patients. Therefore, several researchers prefer to use 1PP to show the experimental materials in the field of empathy for pain (Avenanti et al., 2009; Avenanti, Bueti, Galati, & Aglioti, 2005; Fan & Han, 2008; Gonzalez‐Liencres, Breidenstein, Wolf, & Brüne, 2016; Gu et al., 2010, 2015; Gu & Han, 2007; Gu, Hof, et al., 2013; Jackson et al., 2005; Yao et al., 2016). However, Vistoli et al. also illustrated that the high‐level cognitive processes involved in understanding the emotions of others could be influenced as early as the perceptual level of information processing (Vistoli et al., 2016). Another limitation is the small sample size (*n* < 30 participants). Future studies should consider a bigger sample to explore this issue of visual perspective.

Taken together, the findings of the present study indicated that individual variability in the trait empathy ability were related to volumetric differences and state activities of the AI. An asymmetry existed between the right and left AI between the affective and cognitive empathy. This implied that empathy may be multifaceted and that structural and functional variation in the AI may facilitate self or other related empathic processes in different ways. These findings may be important, because they help to understand how the brain extracts and processes information about other people's pain, which is influenced by individual variability in trait empathy. When we use noninvasive brain stimulation methods to assist in the treatment of empathy‐impaired mental disorders, individual differences in trait empathy might constrain the efficacy of brain stimulation in specific areas. Further studies are needed to combine brain stimulation and fMRI, which will generate new knowledge in functional connectivity changes in empathy for pain in detail, through complementary invasive procedures, clarifying mechanism and improving the therapeutic application of brain stimulation, as well as improving interpretation of the relationship between fMRI data and the personality traits.

## CONFLICT OF INTEREST

The authors declare no conflicts of interest.

## Data Availability

The data that support the findings of this study are available from the corresponding author upon reasonable request.

## References

[hbm24858-bib-0001] Allen, M. , Frank, D. , Glen, J. C. , Fardo, F. , Callaghan, M. F. , & Rees, G. (2017). Insula and somatosensory cortical myelination and iron markers underlie individual differences in empathy. Scientific Reports, 7, 43316. 28256532 10.1038/srep43316 PMC5335674

[hbm24858-bib-0002] Avenanti, A. , Bueti, D. , Galati, G. , & Aglioti, S. M. (2005). Transcranial magnetic stimulation highlights the sensorimotor side of empathy for pain. Nature Neuroscience, 8, 955–960. 15937484 10.1038/nn1481

[hbm24858-bib-0003] Avenanti, A. , Minio‐Paluello, I. , Bufalari, I. , & Aglioti, S. M. (2009). The pain of a model in the personality of an onlooker: Influence of state‐reactivity and personality traits on embodied empathy for pain. NeuroImage, 44, 275–283. 10.1016/j.neuroimage.2008.08.001 18761092

[hbm24858-bib-0004] Banissy, M. J. , Kanai, R. , Walsh, V. , & Rees, G. (2012). Inter‐individual differences in empathy are reflected in human brain structure. NeuroImage, 62, 2034–2039. 22683384 10.1016/j.neuroimage.2012.05.081 PMC3778747

[hbm24858-bib-0005] Basso, F. , Petit, O. , Le Bellu, S. , Lahlou, S. , Cancel, A. , & Anton, J. L. (2018). Taste at first (person) sight: Visual perspective modulates brain activity implicitly associated with viewing unhealthy but not healthy foods. Appetite, 128, 242–254. 29906489 10.1016/j.appet.2018.06.009

[hbm24858-bib-0006] Bernhardt, B. C. , & Singer, T. (2012). The neural basis of empathy. Annual Review of Neuroscience, 35, 1–23. 10.1146/annurev-neuro-062111-150536 22715878

[hbm24858-bib-0007] Canizales, D. L. , Voisin, J. I. A. , Michon, P.‐E. , Roy, M.‐A. , & Jackson, P. L. (2013). The influence of visual perspective on the somatosensory steady‐state response during pain observation. Frontiers in Human Neuroscience, 7, 849. 24367323 10.3389/fnhum.2013.00849 PMC3856401

[hbm24858-bib-0008] Caria, A. , & de Falco, S. (2015). Anterior insular cortex regulation in autism spectrum disorders. Frontiers in Behavioral Neuroscience, 9, 1–9. 25798096 10.3389/fnbeh.2015.00038 PMC4351628

[hbm24858-bib-0009] Carrera, P. , Oceja, L. , Caballero, A. , Muñoz, D. , López‐Pérez, B. , & Ambrona, T. (2013). I feel so sorry! Tapping the joint influence of empathy and personal distress on helping behavior. Motivation and Emotion, 37, 335–345.

[hbm24858-bib-0010] Chakrabarti, B. , Bullmore, E. , & Baron‐Cohen, S. (2006). Empathizing with basic emotions: Common and discrete neural substrates. Social Neuroscience, 1, 364–384. 18633800 10.1080/17470910601041317

[hbm24858-bib-0011] Chiang, S. K. , Hua, M. S. , Tam, W. C. C. , Chao, J. K. , & Shiah, Y. J. (2014). Developing an alternative Chinese version of the interpersonal reactivity index for normal population and patients with schizophrenia in Taiwan. Brain Impairment, 15, 120–131.

[hbm24858-bib-0012] Coll, M. P. , Viding, E. , Rütgen, M. , Silani, G. , Lamm, C. , Catmur, C. , & Bird, G. (2017). Are we really measuring empathy? Proposal for a new measurement framework. Neuroscience and Biobehavioral Reviews, 83, 132–139. 29032087 10.1016/j.neubiorev.2017.10.009

[hbm24858-bib-0013] Couto, B. , Sedeño, L. , Sposato, L. A. , Sigman, M. , Riccio, P. M. , Salles, A. , … Ibanez, A. (2013). Insular networks for emotional processing and social cognition: Comparison of two case reports with either cortical or subcortical involvement. Cortex, 49, 1420–1434. 23036522 10.1016/j.cortex.2012.08.006

[hbm24858-bib-0014] Cox, C. L. , Uddin, L. Q. , Di Martino, A. , Castellanos, F. X. , Milham, M. P. , & Kelly, C. (2012). The balance between feeling and knowing: Affective and cognitive empathy are reflected in the brain's intrinsic functional dynamics. Social Cognitive and Affective Neuroscience, 7, 727–737. 21896497 10.1093/scan/nsr051 PMC3427869

[hbm24858-bib-0015] Craig, A. D. (2002a). Opinion: How do you feel? Interoception: The sense of the physiological condition of the body. Nature Reviews, 3, 655–666. 10.1038/nrn894 12154366

[hbm24858-bib-0016] Craig, A. D. (2003). Interoception: The sense of the physiological condition of the body. Current Opinion in Neurobiology, 13, 500–505. 12965300 10.1016/s0959-4388(03)00090-4

[hbm24858-bib-0017] Craig, A. D. (2009). How do you feel—Now? The anterior insula and human awareness. Nature Reviews. Neuroscience, 10(1), 59–70. 19096369 10.1038/nrn2555

[hbm24858-bib-0018] Craig, A. D. B. (2002b). How do you feel? Interoception: The sense of the physiological condition of the body. Nature Reviews. Neuroscience, 3, 655–666. 12154366 10.1038/nrn894

[hbm24858-bib-0019] Craig, A. D. B. (2011). Significance of the insula for the evolution of human awareness of feelings from the body. Annals of the New York Academy of Sciences, 1225, 72–82. 21534994 10.1111/j.1749-6632.2011.05990.x

[hbm24858-bib-0020] Critchley, H. D. , Wiens, S. , Rotshtein, P. , Öhman, A. , & Dolan, R. J. (2004). Neural systems supporting interoceptive awareness. Nature Neuroscience, 7, 189–195. 14730305 10.1038/nn1176

[hbm24858-bib-0021] Danziger, N. , Faillenot, I. , & Peyron, R. (2009). Can we share a pain we never felt? Neural correlates of empathy in patients with congenital insensitivity to pain. Neuron, 61, 203–212. 19186163 10.1016/j.neuron.2008.11.023

[hbm24858-bib-0022] Davis, M. H. (1983). Measuring individual differences in empathy: Evidence for a multidimensional approach. Journal of Personality and Social Psychology, 44, 113–126.

[hbm24858-bib-0023] Decety, J. , Jackson, P. L. , & Brunet, E. (2007). The cognitive neuropsychology of empathy In T. F. D. Farrow & P. W. R. Woodruff (Eds.), Empathy in mental illness. Cambridge, UK: Cambridge University Press.

[hbm24858-bib-0024] Driscoll, D. M. , Dal Monte, O. , Solomon, J. , Krueger, F. , & Grafman, J. (2012). Empathic deficits in combat veterans with traumatic brain injury: A voxel‐based lesion‐symptom mapping study. Cognitive and Behavioral Neurology, 25, 160–166. 23277137 10.1097/WNN.0b013e318280cf4e

[hbm24858-bib-0025] Duerden, E. G. , Arsalidou, M. , Lee, M. , & Taylor, M. J. (2013). Lateralization of affective processing in the insula. NeuroImage, 78, 159–175. 23587690 10.1016/j.neuroimage.2013.04.014

[hbm24858-bib-0026] Ebisch, S. J. H. , Gallese, V. , Willems, R. M. , Mantini, D. , Groen, W. B. , Romani, G. L. , … Bekkering, H. (2011). Altered intrinsic functional connectivity of anterior and posterior insula regions in high‐functioning participants with autism spectrum disorder. Human Brain Mapping, 32, 1013–1028. 20645311 10.1002/hbm.21085 PMC6870194

[hbm24858-bib-0027] Enzi, B. , Amirie, S. , & Brüne, M. (2016). Empathy for pain‐related dorsolateral prefrontal activity is modulated by angry face perception. Experimental Brain Research, 234, 3335–3345. 27447790 10.1007/s00221-016-4731-4

[hbm24858-bib-0028] Eres, R. , Decety, J. , Louis, W. R. , & Molenberghs, P. (2015). Individual differences in local gray matter density are associated with differences in affective and cognitive empathy. NeuroImage, 117, 305–310. 26008886 10.1016/j.neuroimage.2015.05.038

[hbm24858-bib-0029] Ernst, J. , Northoff, G. , Böker, H. , Seifritz, E. , & Grimm, S. (2013). Interoceptive awareness enhances neural activity during empathy. Human Brain Mapping, 34, 1615–1624. 22359353 10.1002/hbm.22014 PMC6869919

[hbm24858-bib-0030] Fan, Y. , Duncan, N. W. , de Greck, M. , & Northoff, G. (2011). Is there a core neural network in empathy? An fMRI based quantitative meta‐analysis. Neuroscience and Biobehavioral Reviews, 35, 903–911. 10.1016/j.neubiorev.2010.10.009 20974173

[hbm24858-bib-0031] Fan, Y. , & Han, S. (2008). Temporal dynamic of neural mechanisms involved in empathy for pain: An event‐related brain potential study. Neuropsychologia, 46, 160–173. 17825852 10.1016/j.neuropsychologia.2007.07.023

[hbm24858-bib-0032] Forman, S. D. , Cohen, J. D. , Fitzgerald, M. , Eddy, W. F. , Mintun, M. A. , & Noll, D. C. (1995). Improved assessment of significant activation in functional magnetic resonance imaging (fMRI): Use of a cluster‐size threshold. Magnetic Resonance in Medicine, 33, 636–647. 7596267 10.1002/mrm.1910330508

[hbm24858-bib-0033] Gonzalez‐Liencres, C. , Breidenstein, A. , Wolf, O. T. , & Brüne, M. (2016). Sex‐dependent effects of stress on brain correlates to empathy for pain. International Journal of Psychophysiology, 105, 47–56. 27150848 10.1016/j.ijpsycho.2016.04.011

[hbm24858-bib-0034] Gu, X. , Eilam‐Stock, T. , Zhou, T. , Anagnostou, E. , Kolevzon, A. , Soorya, L. , … Fan, J. (2015). Autonomic and brain responses associated with empathy deficits in autism spectrum disorder. Human Brain Mapping, 36, 3323–3338. 25995134 10.1002/hbm.22840 PMC4545680

[hbm24858-bib-0035] Gu, X. , & Han, S. (2007). Attention and reality constraints on the neural processes of empathy for pain. NeuroImage, 36, 256–267. 17400480 10.1016/j.neuroimage.2007.02.025

[hbm24858-bib-0036] Gu, X. , Hof, P. R. , Friston, K. J. , & Fan, J. (2013). Anterior insular cortex and emotional awareness. The Journal of Comparative Neurology, 521, 3371–3388. 23749500 10.1002/cne.23368 PMC3999437

[hbm24858-bib-0037] Gu, X. , Liu, X. , Van Dam, N. T. , Hof, P. R. , & Fan, J. (2013). Cognition‐emotion integration in the anterior insular cortex. Cerebral Cortex, 23, 20–27. 22275476 10.1093/cercor/bhr367 PMC3513949

[hbm24858-bib-0038] Gu, X. , Liu, X. , Guise, K. G. , Naidich, T. P. , Hof, P. R. , & Fan, J. (2010). Functional dissociation of the frontoinsular and anterior cingulate cortices in empathy for pain. The Journal of Neuroscience, 30, 3739–3744. 10.1523/JNEUROSCI.4844-09.2010 20220007 PMC2845539

[hbm24858-bib-0039] Harris, L. J. (1975). Spatial direction and grammatical form of instructions affect the solution of spatial problems. Memory & Cognition, 3, 329–334. 21287082 10.3758/BF03212920

[hbm24858-bib-0040] Hein, G. , Engelmann, J. B. , Vollberg, M. C. , & Tobler, P. N. (2016). How learning shapes the empathic brain. Proceedings of the National Academy of Sciences, 113, 80–85. 10.1073/pnas.1514539112 PMC4711838 26699464

[hbm24858-bib-0041] Hillis, A. E. (2014). Inability to empathize: Brain lesions that disrupt sharing and understanding another's emotions. Brain, 137, 981–997. 24293265 10.1093/brain/awt317 PMC3959550

[hbm24858-bib-0042] Hooley, J. M. , & Delgado, M. L. (2001). Pain insensitivity in the relatives of schizophrenia patients. Schizophrenia Research, 47, 265–273. 11278144 10.1016/s0920-9964(00)00064-5

[hbm24858-bib-0043] Jabbi, M. , Swart, M. , & Keysers, C. (2007). Empathy for positive and negative emotions in the gustatory cortex. NeuroImage, 34, 1744–1753. 17175173 10.1016/j.neuroimage.2006.10.032

[hbm24858-bib-0044] Jackson, P. L. , Meltzoff, A. N. , & Decety, J. (2005). How do we perceive the pain of others? A window into the neural processes involved in empathy. NeuroImage, 24, 771–779. 15652312 10.1016/j.neuroimage.2004.09.006

[hbm24858-bib-0045] Kanai, R. , & Rees, G. (2011). The structural basis of inter‐individual differences in human behaviour and cognition. Nature Reviews. Neuroscience, 12, 231–242 Retrieved from http://www.ncbi.nlm.nih.gov/pubmed/21407245 21407245 10.1038/nrn3000

[hbm24858-bib-0046] Kao, M.‐H. (2014). Recent developments in optimal experimental designs for functional magnetic resonance imaging. World Journal of Radiology, 6(7), 437–445. 25071884 10.4329/wjr.v6.i7.437 PMC4109095

[hbm24858-bib-0047] Kessler, K. , & Thomson, L. A. (2010). The embodied nature of spatial perspective taking: Embodied transformation versus sensorimotor interference. Cognition, 114, 72–88. 19782971 10.1016/j.cognition.2009.08.015

[hbm24858-bib-0048] Keysers, C. , & Gazzola, V. (2014). Dissociating the ability and propensity for empathy. Trends in Cognitive Sciences, 18, 163–166. 10.1016/j.tics.2013.12.011 24484764 PMC4560165

[hbm24858-bib-0049] Kober, H. , Barrett, L. F. , Joseph, J. , Bliss‐moreau, E. , Lindquist, K. , & Wager, T. D. (2008). Functional grouping and cortical–subcortical interactions in emotion. NeuroImage, 42, 998–1031. 18579414 10.1016/j.neuroimage.2008.03.059 PMC2752702

[hbm24858-bib-0050] Lamm, C. , Decety, J. , & Singer, T. (2011). Meta‐analytic evidence for common and distinct neural networks associated with directly experienced pain and empathy for pain. NeuroImage, 54, 2492–2502. 10.1016/j.neuroimage.2010.10.014 20946964

[hbm24858-bib-0051] Leigh, R. , Oishi, K. , Hsu, J. , Lindquist, M. , Gottesman, R. F. , Jarso, S. , … Hillis, A. E. (2013). Acute lesions that impair affective empathy. Brain, 136, 2539–2549. 23824490 10.1093/brain/awt177 PMC3722353

[hbm24858-bib-0052] Loggia, M. L. , Mogil, J. S. , & Catherine Bushnell, M. (2008). Empathy hurts: Compassion for another increases both sensory and affective components of pain perception. Pain, 136, 168–176. 17822850 10.1016/j.pain.2007.07.017

[hbm24858-bib-0053] Masten, C. L. , Morelli, S. A. , & Eisenberger, N. I. (2011). An fMRI investigation of empathy for “social pain” and subsequent prosocial behavior. NeuroImage, 55, 381–388. 21122817 10.1016/j.neuroimage.2010.11.060

[hbm24858-bib-0054] Melchers, M. , Montag, C. , Reuter, M. , Spinath, F. M. , & Hahn, E. (2016). How heritable is empathy? Differential effects of measurement and subcomponents. Motivation and Emotion, 40, 720–730.

[hbm24858-bib-0055] Mutschler, I. , Reinbold, C. , Wankerl, J. , Seifritz, E. , & Ball, T. (2013). Structural basis of empathy and the domain general region in the anterior insular cortex. Frontiers in Human Neuroscience, 7, 1–9. 10.3389/fnhum.2013.00177/abstract PMC3648769 23675334

[hbm24858-bib-0056] Nieuwenhuys, R. (2012). The insular cortex. A review. Progress in Brain Research, 195, 123–163. 22230626 10.1016/B978-0-444-53860-4.00007-6

[hbm24858-bib-0057] Paciello, M. , Fida, R. , Cerniglia, L. , Tramontano, C. , & Cole, E. (2013). High cost helping scenario: The role of empathy, prosocial reasoning and moral disengagement on helping behavior. Personality and Individual Differences, 55, 3–7.

[hbm24858-bib-0058] Pfeifer, J. H. , Iacoboni, M. , Mazziotta, J. C. , & Dapretto, M. (2008). Mirroring others' emotions relates to empathy and interpersonal competence in children. NeuroImage, 39, 2076–2085. 18082427 10.1016/j.neuroimage.2007.10.032 PMC3840169

[hbm24858-bib-0059] Rankin, K. P. , Gorno‐Tempini, M. L. , Allison, S. C. , Stanley, C. M. , Glenn, S. , Weiner, M. W. , & Miller, B. L. (2006). Structural anatomy of empathy in neurodegenerative disease. Brain, 129, 2945–2956. 17008334 10.1093/brain/awl254 PMC2562652

[hbm24858-bib-0060] Sassa, Y. , Taki, Y. , Takeuchi, H. , Hashizume, H. , Asano, M. , Asano, K. , … Kawashima, R. (2012). The correlation between brain gray matter volume and empathizing and systemizing quotients in healthy children. NeuroImage, 60, 2035–2041. 22369996 10.1016/j.neuroimage.2012.02.021

[hbm24858-bib-0061] Singer, T. , Seymour, B. , O'Doherty, J. , Kaube, H. , Dolan, R. J. , & Frith, C. D. (2004). Empathy for pain involves the affective but not sensory components of pain. Science (80‐), 303, 1157–1162. 10.1126/science.1093535 14976305

[hbm24858-bib-0062] Singh, M. K. , Giles, L. L. , & Nasrallah, H. A. (2006). Pain insensitivity in schizophrenia: Trait or state marker? Journal of Psychiatric Practice, 12, 90–102. 16728905 10.1097/00131746-200603000-00004

[hbm24858-bib-0063] Švegar, D. , Antulov, R. , Tkalčić, M. , & Antončić, I. (2016). Lesions of left basal ganglia and insula structures impair executive functions but not emotion recognition: A case report. Brain Impairment, 17, 233–241.

[hbm24858-bib-0064] Takeuchi, H. , Taki, Y. , Sassa, Y. , Hashizume, H. , Sekiguchi, A. , Fukushima, A. , & Kawashima, R. (2014). Regional gray matter volume is associated with empathizing and systemizing in young adults. PLoS One, 9, e84782. 24409308 10.1371/journal.pone.0084782 PMC3883687

[hbm24858-bib-0065] Thomas, J. (2013). Association of personal distress with burnout, compassion fatigue, and compassion satisfaction among clinical social workers. Journal of Social Service Research, 39, 365–379.

[hbm24858-bib-0066] Timmers, I. , Park, A. L. , Fischer, M. D. , Kronman, C. A. , Heathcote, L. C. , Hernandez, J. M. , & Simons, L. E. (2018). Is empathy for pain unique in its neural correlates? A meta‐analysis of neuroimaging studies of empathy. Frontiers in Behavioral Neuroscience, 12, 1–12. 30542272 10.3389/fnbeh.2018.00289 PMC6277791

[hbm24858-bib-0067] Vachon‐Presseau, E. , Roy, M. , Martel, M. O. , Albouy, G. , Chen, J. , Budell, L. , … Rainville, P. (2012). Neural processing of sensory and emotional‐communicative information associated with the perception of vicarious pain. NeuroImage, 63, 54–62. 22732556 10.1016/j.neuroimage.2012.06.030

[hbm24858-bib-0068] Vistoli, D. , Achim, A. M. , Lavoie, M. A. , & Jackson, P. L. (2016). Changes in visual perspective influence brain activity patterns during cognitive perspective‐taking of other people's pain. Neuropsychologia, 85, 327–336. 27012986 10.1016/j.neuropsychologia.2016.03.020

[hbm24858-bib-0069] Wager, T. D. , Phan, K. L. , Liberzon, I. , & Taylor, S. F. (2003). Valence, gender, and lateralization of functional brain anatomy in emotion: A meta‐analysis of findings from neuroimaging. NeuroImage, 19, 513–531. 12880784 10.1016/s1053-8119(03)00078-8

[hbm24858-bib-0070] World Medical Association . (2013). World Medical Association declaration of Helsinki: Ethical principles for medical research involving human subjects. JAMA—Journal of the American Medical Association, 310(20), 2191–2194. 10.1001/jama.2013.281053 24141714

[hbm24858-bib-0071] Wu, L. , Kirmse, U. , Flaisch, T. , Boiandina, G. , Kenter, A. , & Schupp, H. T. (2017). Empathy, pain and attention: Cues that predict pain stimulation to the partner and the self capture visual attention. Frontiers in Human Neuroscience, 11, 465. 28979199 10.3389/fnhum.2017.00465 PMC5611362

[hbm24858-bib-0072] Wylie, K. P. , & Tregellas, J. R. (2010). The role of the insula in schizophrenia. Schizophrenia Research, 123, 93–104. 20832997 10.1016/j.schres.2010.08.027 PMC2957503

[hbm24858-bib-0073] Yao, S. , Becker, B. , Geng, Y. , Zhao, Z. , Xu, X. , Zhao, W. , … Kendrick, K. M. (2016). Voluntary control of anterior insula and its functional connections is feedback‐independent and increases pain empathy. NeuroImage, 130, 230–240. 26899786 10.1016/j.neuroimage.2016.02.035

[hbm24858-bib-0074] Zaki, J. , & Ochsner, K. (2012). The neuroscience of empathy: Progress, pitfalls and promise. Nature Neuroscience, 15, 675–680. 22504346 10.1038/nn.3085

[hbm24858-bib-0075] Zhang, F. , Dong, Y. , Wang, K. , Zhan, Z. , & Xie, L. (2010). Chinese version interpersonal reactivity index (IRI‐C): A study of reliability and validity. Chinese Journal of Clinical Psychology, 18, 155–157.

[hbm24858-bib-0076] Zhu, Y. , Xie, X. , Zhong, S. , Yang, J. , Zhou, H. , Gu, L. , … Wang, C. (2018). Brain structural and functional substrates of personal distress in empathy. Frontiers in Behavioral Neuroscience, 12, 1–7. 29867397 10.3389/fnbeh.2018.00099 PMC5962755

